# Primary angioplasty/stenting versus mechanical thrombectomy as the initial approach for underlying ICAD-LVO: a multicenter retrospective cohort study

**DOI:** 10.3389/fneur.2026.1761891

**Published:** 2026-01-14

**Authors:** Shuai Mi, Lin Chen, Xianhua Hou, Yuxuan He, Qu Liu, Tao Lv, Liangbo Kong, Li Wang, Zhenhua Zhou

**Affiliations:** 1 Department of Neurology, The First Affiliated Hospital (Southwest Hospital), Army Medical University, Chongqing, China; 2 Department of Neurology, Zigong Third People's Hospital, Zigong, Sichuan, China

**Keywords:** angioplasty, ICAD-LVO, initial approach, mechanical Thrombectomy (MT), stenting

## Abstract

**Objective:**

Intracranial atherosclerotic disease-related large vessel occlusion (ICAD-LVO) is a prevalent stroke subtype among Asian populations. Characterized by dynamic intraprocedural reocclusion and underlying stenotic pathology, this condition poses distinct challenges for acute endovascular management. Contemporary guidelines universally recommend mechanical thrombectomy (MT) as first-line recanalization therapy for large vessel occlusion (LVO); however such recommendations derive predominantly from studies without etiological stratification. Consequently, optimal initial endovascular strategies for ICAD-LVO remain undefined despite its epidemiological significance in Asian cohorts.

**Methods:**

In this multicenter retrospective cohort study, 161 patients with underlying ICAD-LVO who underwent endovascular therapy (June 2022–December 2024) were stratified by initial strategy: angioplasty/stenting (AS group, *n* = 94) or mechanical thrombectomy (MT group, *n* = 67). The primary outcome was 90-day functional independence (modified Rankin Scale [mRS] score 0–2). Secondary outcomes included successful recanalization (mTICI 2b–3), symptomatic intracranial hemorrhage (SICH), and mortality.

**Results:**

Among 161 included patients (94 AS vs. 67 MT), baseline characteristics were balanced except for a higher prevalence of hyperlipidemia (*p* = 0.041), progressive stroke (*p* < 0.001), and tirofiban administration (*p* = 0.043) in the AS group. The initial AS approach achieved significantly higher rates of functional independence (63.8% vs. 47.8%; adjusted OR = 2.886, 95% CI: 1.290–6.736, *p* = 0.011) and reduced the need for rescue therapy (24.5% vs. 55.2%). Rates of successful recanalization (96.8% vs. 91.0%, *p* = 0.155), SICH (13.8% vs. 13.4%, *p* = 0.985), and 90-day mortality (14.9% vs. 13.4%, *p* = 0.784) did not differ significantly.

**Conclusion:**

In this multicenter retrospective cohort study, primary angioplasty/stenting was associated with superior clinical efficacy compared to mechanical thrombectomy as the initial approach for underlying ICAD-LVO. This approach showed higher rates of 90-day functional independence while maintaining a comparable safety profile. These findings support the concept of etiology-specific endovascular strategies; however, this approach requires confirmation in prospective randomized controlled trials.

## Introduction

Acute ischemic stroke (AIS) due to large vessel occlusion (LVO) results in severe neurological deficits and imposes a substantial healthcare burden (1). Endovascular therapy (EVT), particularly mechanical thrombectomy (MT), has revolutionized stroke management by restoring cerebral blood flow and is now the standard first-line treatment for eligible patients (2, 3). However, intracranial atherosclerotic disease (ICAD) is a frequent cause of LVO in Asian populations (4). ICAD-related LVO (ICAD-LVO) has a distinct pathophysiological process, involving an underlying stenotic artery with local thrombus formation. This leads to more complex treatments and higher rates of intraprocedural vessel reocclusion compared to strokes caused by cardioembolic occlusions (5–7).

Current evidence for treating ICAD-LVO is limited. Major stroke trials recommend MT as the initial approach. They suggest using balloon angioplasty or stenting only as rescue therapy when MT fails (8–11). But these trials did not separate stroke causes. Their advice may not fit ICAD-LVO’s unique challenges. ICAD-LVO procedures often require rescue therapy. Studies show 35–51% of cases need additional angioplasty or stenting after initial MT (12, 13). This high rescue rate underscores the need for better initial strategies. Some experts now propose treating the underlying narrowing directly from the start. Levy et al. first tested this method of primary angioplasty/stenting (AS). In their small cohort of 20 patients, they found that primary AS was associated with a 60% rate of functional independence (modified Rankin Scale [mRS] ≤ 3) and a 5% rate of symptomatic intracranial hemorrhage (SICH) (14). While promising, this single-center experience was underpowered to draw definitive conclusions.

We still lack clear evidence about the best initial approach for ICAD-LVO. To address this gap, we conducted a multicenter study comparing primary AS versus MT as the initial treatment for ICAD-LVO patients.

## Methods

### Study design and patient selection

This multicenter, retrospective cohort study analyzed consecutive patients with AIS due to underlying ICAD-LVO who underwent EVT between June 2022 and December 2024. The study was conducted at two comprehensive stroke centers in China: The First Affiliated Hospital of Army Medical University and Zigong Third People’s Hospital of Sichuan Province.

**Figure 1:**
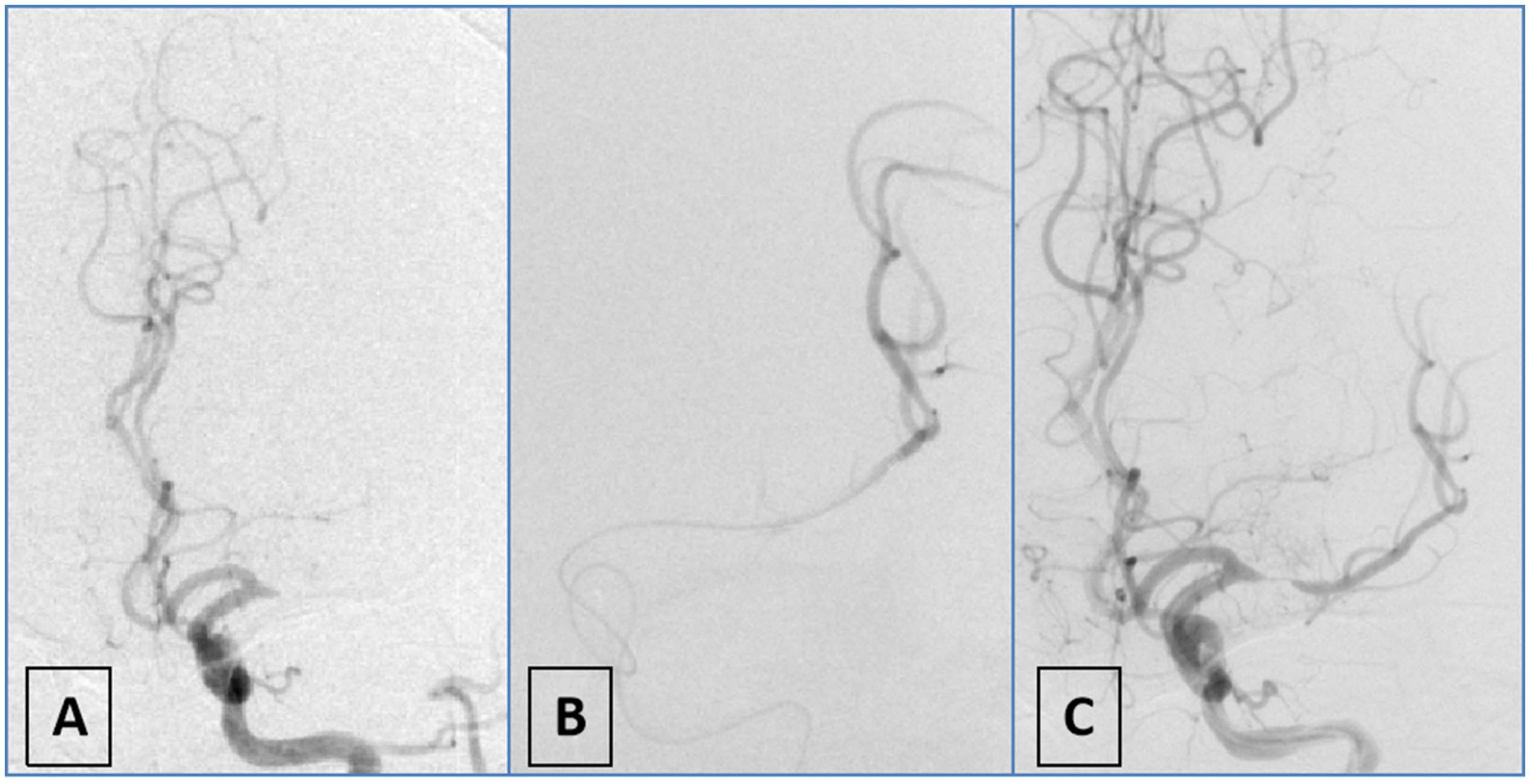
Schematic diagram of first-pass effect positivity. **(A)** DSA angiography demonstrates occlusion of the M1 segment of the left middle cerebral artery; **(B)** the microwire and microcatheter traverse the lesion to reach the distal vasculature, confirming intraluminal positioning; **(C)** repeat angiography after partial withdrawal of the microcatheter reveals severe stenosis in the M1 segment of the left middle cerebral artery.

**Figure 2:**
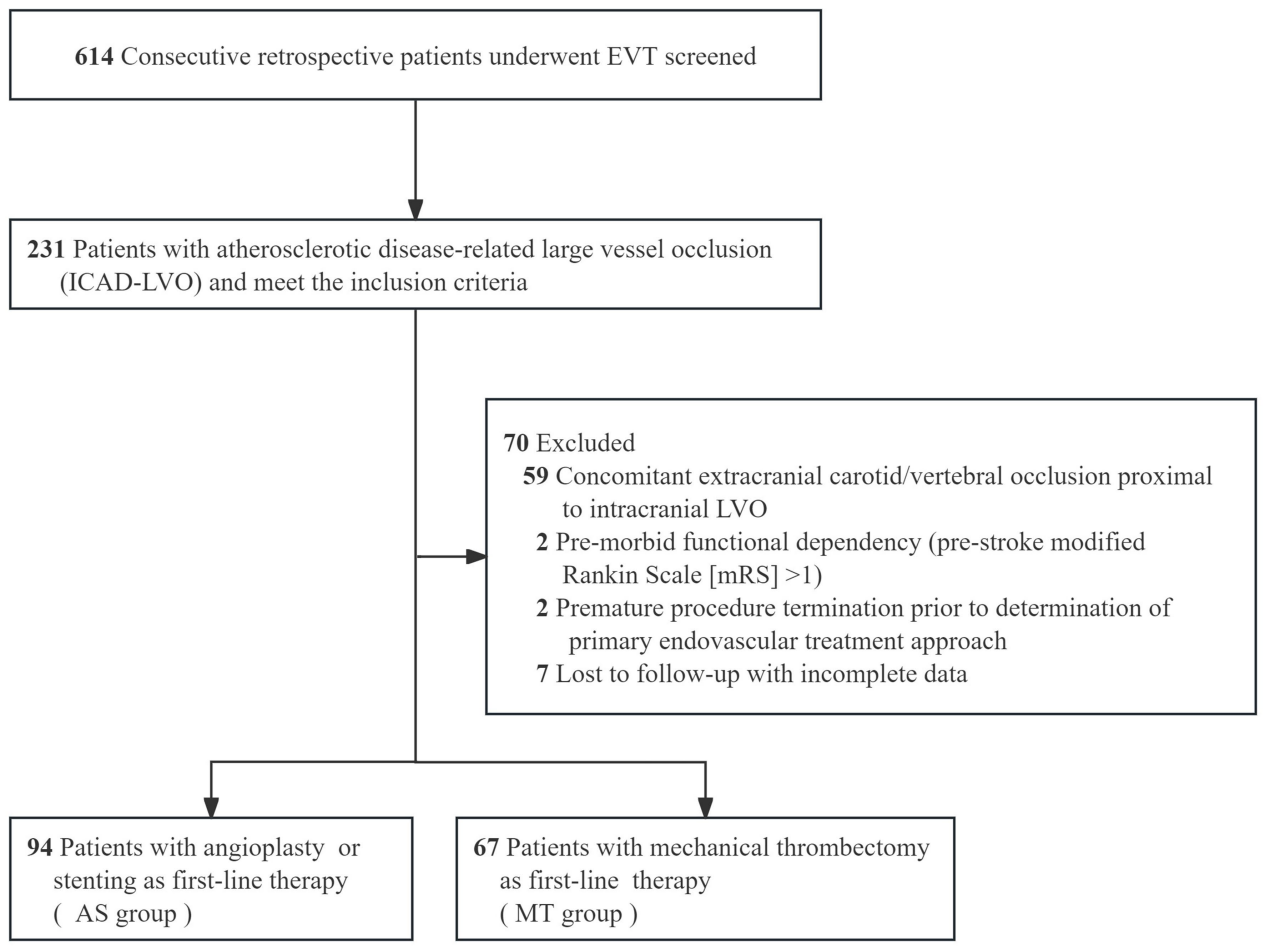
The flow diagram of the patient inclusion process in the study.

Patients were included if they were ≥ 18 years of age, had no evidence of intracranial hemorrhage on baseline non-contrast CT or MRI, and had an intracranial LVO confirmed by CTA, MRA, or DSA. Occlusions of the vertebral artery V4 segment were included only in the presence of contralateral vertebral artery hypoplasia or occlusion. The underlying etiology of ICAD-LVO was determined using a positive microcatheter first-pass effect (15) (Figure 1). Additional inclusion criteria consisted of a baseline National Institutes of Health Stroke Scale [NIHSS] score ≥6, indicating moderate to severe deficits, and an onset-to-puncture time within 24 h. Key exclusion criteria covered procedural termination before implementation of the initial approach, pre-stroke functional dependence (defined as mRS > 1), and the presence of ≥70% stenosis or occlusion proximal to the occluded vessel suggestive of an artery-to-artery embolism mechanism. We also excluded patients with refractory hypertension (>185/110 mmHg despite medication), severe dysglycemia (blood glucose <2.7 or >22.2 mmol/L), coagulopathy (platelet count <40 × 10^9^/L, aPTT >50 s, or INR > 3), and those with incomplete clinical or angiographic data. These criteria were applied to minimize confounding related to comorbidities, technical variability, and data reliability issues. The final analysis included 161 patients with ICAD-LVO, who were classified into two groups according to the initial endovascular strategy: the AS group and the MT group (Figure 2).

The study protocol received ethical approval from the institutional review boards of both participating hospitals (Approval No. B-KY2025079). Informed consent was waived due to the retrospective design and use of anonymized clinical data. All procedures were conducted in accordance with the ethical principles of the Declaration of Helsinki.

### Diagnosis of underlying ICAD-LVO

Diagnosis of underlying ICAD-LVO in this study was primarily determined through preprocedural clinical data, preprocedural CTA, and intraprocedural DSA imaging. First, suspicion for ICAD-LVO arose if patients had preexisting atherosclerotic risk factors (hypertension; diabetes; smoking) (16, 17) or exhibited trunk occlusion rather than branch occlusion (18). Operators subsequently verified the diagnosis intraprocedurally using the microcatheter first-pass effect (15). To evaluate this effect, the microcatheter was advanced with the microguidewire through the occlusion into the distal patent segment and then withdrawn to a position proximal to the occlusion while the microguidewire remained in place, after which angiography was performed via an intermediate catheter. A positive result was defined as the angiographic demonstration of both transient antegrade flow and an underlying severe stenosis at the occlusion site, a finding indicative of an underlying ICAD-LVO. The absence of flow (persistent occlusion) was considered a negative result, suggestive of an embolic etiology (Figure 1). Diagnosis was made intraoperatively by the operator and prospectively recorded in the database. Subsequently, another neurointerventionalist reviewed the diagnosis. Discrepancies were resolved through unanimous consensus.

### Endovascular therapy

All endovascular procedures were performed under general anesthesia via a femoral artery approach. All operators had ≥10 years of experience in acute stroke neurointervention, and the initial strategies were selected based on individual expertise. All endovascular devices were approved by China’s National Medical Products Administration for intracranial interventional procedures. All patients followed a standardized heparinization protocol: a 3,000-unit intravenous bolus was given immediately after femoral artery puncture, followed by a maintenance dose of 1,000 units per hour. No heparin was administered to patients who had received preoperative intravenous thrombolysis.

Postprocedural management adhered to standardized stroke unit protocols: systolic blood pressure maintained <140 mmHg, serial neurological assessments (every 60 min for 24 h), and individualized secondary prevention. A uniform antiplatelet regimen was administered: continuous intravenous tirofiban infusion for 24 h, transitioning to oral aspirin (100 mg/day) plus clopidogrel (75 mg/day) for 3 months. Intravenous tirofiban was generally initiated immediately after angioplasty or mechanical thrombectomy at a maintenance infusion rate of 0.2–0.25 mg/h. In cases of recurrent occlusion attributed to endothelial injury, an additional intracatheter (intra-arterial) bolus of 0.25–0.5 mg could be administered locally. For patients who had undergone prior intravenous thrombolysis or exhibited contrast extravasation on post-EVT imaging, antiplatelet agents were adjusted—either discontinued or reduced—following multidisciplinary consensus on hemorrhagic transformation risk.

#### AS Group

Operators performed intracranial balloon angioplasty using gradual inflation sustained at 4 atmospheres for 60 s. Immediate post-deflation angiography assessed vessel response, with stent implantation contingent upon operator identification of significant residual stenosis or flow limitation during real-time angiographic evaluation. When suboptimal reperfusion (mTICI grade <2b) occurred, rescue strategies comprised: use of a stent retriever or contact aspiration thrombectomy following initial balloon angioplasty; aspiration thrombectomy within the deployed stent for persistent thrombus; or balloon redilation after stent implantation.

#### MT Group

Operators employed the following two methods: (1) Direct aspiration via placement of a large-bore intermediate catheter at the vascular occlusion site; (2) Deployment of a stent retriever within the thrombus with maintenance of engagement for 60 s, followed by device retrieval under continuous aspiration. Rescue interventions were permitted after 1–3 unsuccessful retrieval attempts and comprised balloon/stent angioplasty for underlying stenosis.

### Outcome assessment

Functional independence at 90 days, defined as mRS scores 0 to 2, served as the primary endpoint. Neurologists prospectively collected outcome data through structured electronic health record abstraction and validated telephone-based mRS assessments. To ensure methodological rigor and reliability, all assessments were performed by neurologists with specialized expertise in stroke outcomes.

Secondary outcomes included successful recanalization (defined as modified Thrombolysis in Cerebral Infarction [mTICI] grade 2b–3) (19), symptomatic intracranial hemorrhage (SICH) within 48 h (classified according to the Heidelberg bleeding classification) (20), and 90-day all-cause mortality.

### Statistical analysis

Data analyses were conducted using SPSS Statistics (version 29.0; IBM). Baseline characteristics were summarized as proportions (%) for categorical variables, and as mean ± standard deviation (SD) or median (interquartile range [IQR]) for continuous variables, based on normality assessed by Shapiro–Wilk tests. Group comparisons utilized chi-square or Fisher’s exact tests for categorical variables, and independent t-tests or Mann–Whitney U tests for continuous variables, as appropriate. Logistic regression models were applied to evaluate associations between first-line recanalization strategies and clinical outcomes. Variables with *p* < 0.10 in univariate analyses alongside prespecified clinical covariates (age, diabetes, baseline NIHSS) were included in adjusted models. Results are presented as crude and adjusted odds ratios (ORs) with 95% confidence intervals (CIs). Multicollinearity was verified using variance inflation factors (VIF < 2.0), and model calibration was confirmed via Hosmer-Lemeshow tests (*p* > 0.05). Statistical significance was defined as a two-tailed *p* < 0.05.

## Results

### Study population and baseline clinical characteristics

Among 614 consecutive patients undergoing emergency EVT for LVO, 37.6% (231/614) were diagnosed with underlying ICAD-LVO. After applying the inclusion and exclusion criteria, the final analysis included 161 patients (107 males and 54 females) with a mean age of 67 years (range, 34–96 years), as detailed in Figure 2. Baseline demographic and clinical characteristics are summarized in Table 1.

**Table 1:** Demographic and clinical characteristics of the patients.

Characteristic	All patients (*N* = 161)	AS group (*N* = 94)	MT group (*N* = 67)	*p* value
Age, mean(±SD)	66.5 ± 12.4	65.9 ± 11.8	67.3 ± 13.2	0.471
Male	107 (66.46)	65 (69.15)	42 (62.69)	0.392
Medical history				
Hypertension	117 (72.7)	69 (73.4)	48 (71.6)	0.805
Hyperlipidemia	68 (42.2)	46 (48.9)	22 (32.8)	0.041
Diabetes	57 (35.4)	35 (37.2)	22 (32.8)	0.565
Smoking	68 (42.2)	38 (40.4)	30 (44.8)	0.582
Alcohol	57 (35.4)	36 (38.3)	21 (31.3)	0.363
Atrialfibrillation	9 (5.6)	5 (5.3)	4 (6.0)	0.859
Previous stroke/TIA	14 (8.7)	11 (11.7)	3 (4.5)	0.109
Lesion location				
Anterior circulation	126 (78.3)	73 (77.7)	53 (79.1)	0.827
ICA C7	24 (14.9)	15 (16.0)	9 (13.4)	
MCA M1-M2	94 (58.4)	53 (56.4)	41 (61.2)	
ACA A1	8 (5.0)	5 (5.3)	3 (4.5)	
Posterior circulation	35 (21.7)	21 (22.3)	14 (20.9)	0.827
BA	27 (16.7)	16 (17.0)	11 (16.4)	
VA V4	8 (5.0)	5 (5.3)	3 (4.5)	
Progressive stroke**	64 (39.8)	48 (51.1)	16 (23.9)	<0.001
Wakeupstroke	16 (9.9)	10 (10.6)	6 (9.0)	0.725
Intravenous thrombolysis	21 (13.0)	16 (17.0)	5 (7.5)	0.076
Tirofiban	142 (88.2)	87 (92.6)	55(82.1)	0.043
NIHSS, median[IQR]#	12.0 [8.0, 18.0]	11.0 [8.0, 18.0]	12.0 [8.0,18.0]	0.847
Stroke-to-puncture time in hour, median [IQR]	7.2 [5.2, 11.3]	7.6 [4.9, 13.2]	6.5 [5.2,9.0]	0.227
Puncture to recanalisation time in minute, median [IQR]§	65.0 [46.0, 97.0]	65.0 [47.0, 100.0]	64.0 [45.0,90.0]	0.482
ASPECT, median [IQR]*	7.0 [6.0, 8.0]	8.0[6.0, 8.0]	7.0 [6.0,8.0]	0.670

Data are *n* (%) unless otherwise stated. SD, standard deviation; TIA, transient ischemic attack; IQR, interquartile range; ICA, internal carotid artery; MCA, middle cerebral artery; ACA, anterior cerebral artery; BA, basilar artery; VA, vertebral artery; NIHSS, National Institutes of Health Stroke Scale; ASPECT, Alberta Stroke Program Early CT Score. **Progressive stroke is defined as an increase in NIHSS score of ≥4 points. #NIHSS range from 0 to 42, with higher scores indicating more severe neurologic deficits. §The time of the therapy completion is regarded as the reperfusion time for patients whose reperfusion failed. *ASPECT score is a measure of the extent of the stroke. Scores range from 0 to 10, with higher scores indicating fewer early ischemic changes.

Most baseline characteristics were well balanced between the two groups, including age, sex, occlusion sites, wake-up stroke, intravenous thrombolysis, baseline NIHSS and Alberta Stroke Program Early CT Score [ASPECTS], and procedural time metrics (all *p* > 0.05). However, the AS group had significantly higher rates of hyperlipidemia (48.9% vs. 32.8%, *p* = 0.041), progressive stroke onset (51.1% vs. 23.9%, *p* < 0.001), and tirofiban administration (92.6% vs. 82.1%, *p* = 0.043).

### Initial and rescue intervention approaches

The technical details of the initial and rescue interventions are presented in Table 2. In the AS group, balloon angioplasty alone was performed in 33 patients (35.1%), while 61 patients (64.9%) received adjunctive stent implantation. In the MT group, initial strategy was aspiration thrombectomy in 24 patients (35.8%) and a combined approach (aspiration with stent retriever) in 43 patients (64.2%).

**Table 2:** Analysis of rescue interventions.

Characteristic	AS group (*N* = 94)	MT group (*N* = 67)
First-line recanalization rate*	69 (73.4)	25 (37.3)
Rescue intervention#	23 (24.5)	37 (55.2)
Stent-retriever/aspiration after balloon	18/23 (78.3)	_
Aspiration after stenting	1/23 (4.3)	_
Balloon re-dilation after stenting	4/23 (17.4)	_
Balloon/stent angioplasty	_	37/37 (100)
Rescue recanalization rate‡	22/23 (95.7)	36/37 (97.3)

Data are *n* (%) unless otherwise stated. *First-line Recitalization was defined as successful vascular recanalization (mTICI ≥2b) achieved by the first-line thrombectomy strategy without rescue interventions, in which the AS group underwent ≤3 balloon inflations and the MT group performed ≤3 retrieval attempts. #Rescue Intervention is defined as taking action after the first-line strategy has been employed at least once (generally no more than three times). ‡The Rescue Recanalization Rate is defined as the proportion of patients achieving successful recanalization among those who received rescue interventions.

Successful initial recanalization without rescue therapy was achieved in 69 patients (73.4%) in the AS group compared to 25 patients (37.3%) in the MT group. Rescue interventions were initiated in 23 patients (24.5%) from the AS group and 37 patients (55.2%) from the MT group. Among patients in the AS group requiring rescue, subsequent strategies included stent-retriever/aspiration after balloon (78.3%, 18/23), aspiration after stenting (4.3%, 1/23), and balloon re-dilation after stenting (17.4%, 4/23). All rescue cases in the MT group received balloon or stent angioplasty (100%, 37/37). Successful rescue recanalization was observed in 22 patients (95.7%) in the AS group and 36 patients (97.3%) in the MT group.

### Primary and safety outcomes

Clinical outcomes are summarized in Table 3. In unadjusted analysis, the AS group demonstrated a higher proportion of functional independence compared to the MT group (63.8% vs. 47.8%, OR = 1.930, 95% CI: 1.020–3.653, *p* = 0.043). The multivariate logistic regression model was adjusted for covariates identified in univariate analyses (baseline NIHSS score, stroke-to-puncture time, and lesion location; threshold: *p* < 0.1) and prespecified clinically relevant confounders, including age, baseline NIHSS score, tirofiban administration, and diabetes mellitus. After adjustment, this association remained statistically significant (adjusted OR = 2.886, 95% CI: 1.290–6.736, *p* = 0.011).

**Table 3:** Univariate and multivariate analysis of clinical outcomes.

Outcome	AS group (*N* = 94)	MT group (*N* = 67)	Unadjusted logistic analysis		Adjusted logistic analysis*	
			OR (95% CI)	*P* value	OR (95% CI)	*P* value
Primary outcome						
mRS score of 0–2 at 90 days	60 (63.8)	32 (47.8)	1.930 (1.020–3.653)	0.043	2.886 (1.290–6.736)	0.011
Secondary outcomes						
Recanalization#	91 (96.8)	61 (91.0)	2.984 (0.719–12.385)	0.132	2.958 (0.703–15.449)	0.155
SICH within 48 h‡	13 (13.8)	9 (13.4)	1.034 (0.415–2.581)	0.942	1.009 (0.391–2.702)	0.985
Death at 90 days	14 (14.9)	9 (13.4)	1.128 (0.457–2.782)	0.794	1.151 (0.425–3.273)	0.784

Data are *n* (%) unless otherwise stated. mRS, modified Rankin Scale score; OR, odds ratio; CI, confidence interval; SICH, symptomatic intracranial haemorrhage. *Adjusted for age, NIHSS, diabetes, lesion location, tirofiban, and stroke-to-puncture time. #Recanalization is defined as immediate postoperative DSA showing a mTICI blood flow grade of >2a. ‡According to the Heidelberg bleeding classification.

No statistically significant differences were observed across secondary outcomes between the AS and MT groups. Recanalization rates were comparable (96.8% vs. 91.0%, *p* = 0.132), with no association identified after adjustment (adjusted OR = 2.958, 95% CI: 0.703–15.449, *p* = 0.155). Similarly, SICH within 48 h (13.8% vs. 13.4%, *p* = 0.942) and all-cause mortality at 90 days (14.9% vs. 13.4%, *p* = 0.794) remained comparable between groups, with adjusted OR of 1.009 (95% CI: 0.391–2.702, *p* = 0.985) and 1.151 (95% CI: 0.425–3.273, *p* = 0.784).

## Discussion

In this multicenter retrospective study, primary AS was associated with superior clinical efficacy compared to MT as an initial approach for ICAD-LVO. Patients treated with initial AS demonstrated significantly higher rates of 90-day functional independence, while exhibiting comparable safety profiles in terms of SICH and mortality. These findings suggest that thrombectomy-first strategies may not be universally optimal for this specific stroke etiology and highlight the potential value of tailoring initial endovascular strategies to the underlying cause.

Atherosclerotic plaque disruption is widely recognized as the primary mechanism underlying thrombus formation in ICAD, leading to cerebrovascular events (21). Rupture of the culprit plaque exposes subendothelial collagen, triggering platelet aggregation and subsequent LVO. Unlike cardioembolic occlusion, ICAD-LVO involves not only acute thrombus but also underlying stenotic atherosclerotic plaque. When thrombus burden is high, MT may represent the preferred strategy for rapid removal. However, in cases with minimal thrombus, MT attempts could fail to retrieve clot material. More importantly, such device manipulation may aggravate endothelial injury and further expose thrombogenic collagen substrates. This process enhances platelet adhesion efficiency and intensifies the thrombotic cascade (22). This pathophysiological mechanism likely explains both the unfavorable outcomes observed in the ANGEL-REBOOT trial when angioplasty was used as rescue therapy after MT and the consistently high reocclusion rates reported in ICAD-LVO (6, 7, 23).

Contrary to prior approaches employing AS as rescue therapy, this study implemented AS as the initial recanalization strategy. Our data indicate that initial AS approach is associated with improved 90-day functional outcomes. Specifically, this strategy achieved immediate recanalization without requiring rescue therapy in 73.4% of cases, which contrasts sharply with the 37.3% success rate of primary MT. Four factors may contribute to the superiority of initial AS approach: (1) Using a positive microcatheter first-pass effect as a diagnostic criterion for underlying ICAD-LVO not only facilitates etiological diagnosis but also effectively identifies patients with low thrombus burden. (2) Initial AS approach enables rapid restoration of vessel patency, which reduces the local concentration of coagulation factors and thereby interrupts the thrombotic cascade. (3) Administration of tirofiban shifts the hemostatic balance by inhibiting platelet aggregation, tipping the equilibrium toward endogenous fibrinolysis. (4) Avoiding mechanical thrombectomy (MT) minimizes direct endothelial trauma, consequently reducing the risk of frequent intraprocedural reocclusion.

While this study supports the use of initial AS as an effective recanalization strategy for underlying ICAD-LVO, several limitations should be acknowledged. First, the diagnosis of underlying ICAD-LVO relied heavily on a positive microcatheter first-pass effect. This criterion may not capture the full spectrum of ICAD-LVO presentations, which limits the generalizability of our conclusions. Second, despite statistical adjustments for key prognostic variables, the non-randomized design carries an inherent risk of residual confounding. Finally, the selection of the initial recanalization strategy was not randomized but depended on the operating physician’s clinical judgment and experience, potentially introducing selection bias.

## Conclusion

Multicenter retrospective analyses suggest that primary AS may provide relatively better clinical outcomes than MT when utilized as the initial approach for ICAD-LVO. This approach was associated with higher rates of 90-day functional independence, while demonstrating comparable safety profiles to MT. The observed clinical differences may be partially attributable to direct treatment of the underlying atherosclerotic lesion, which appears to reduce endothelial trauma associated with repetitive device manipulation. Our findings support the clinical value of etiology-specific interventional strategies. Future randomized controlled trials should validate these results.

## Data Availability

The raw data supporting the conclusions of this article will be made available by the authors, without undue reservation.
